# Supplementary material for: Influence of Polymer Composition on the Controlled Release of Docetaxel: A Comparison of Non-Degradable Polymer Films for Oesophageal Drug-Eluting Stents
Source: Pharmaceutics. 2020 May 11;12(5):444. doi: 10.3390/pharmaceutics12050444 (PMC7284596; doi:10.3390/pharmaceutics12050444)
Supplement: Supplementary file 1 [file pharmaceutics-12-00444-s001.pdf]

pharmaceutics

Supplementary Materials: Influence of Polymer Composition on the Controlled Release of Docetaxel: A Comparison of Non-Degradable Polymer Films for Oesophageal Drug-Eluting Stents

Paris Fouladian, Franklin Afinjuomo, Mohammad Arafat, Amanda Bergamin, Yunmei Song, Anton Blencowe and Sanjay Garg

Table S1. Actual amount of DTX and polymer used in each formulation.

Formulation	Actual Amount of DTX (mg)	Actual Amount of Polymer (mg)
$PEVA_1$	4.04	400
$PEVA_5$	21.05	400
$PEVA_{10}$	44.44	400
PU_1	3.88	385
PU_5	20.26	385
PU_{10}	42.44	385
Psi_1	2.80	278
Psi_5	14.63	278
Psi_{10}	30.9	278

Table S2. Equations for release kinetics models.

Mathematical Models	Equation
Zero order	$Q_t = Q_0+K_0t$
First order	$\ln Q_t = \ln Q_0+K_1t$
Higuchi	$Qt = KH$ t ½
Hixson-Crowell	Q_0⅓ − Q $_t$⅓= Ks t
Korsmeyer-Peppas	$Q_t/Q\infty = Kktn$

Qt: amount of drug released in time t; $Q0$: initial amount of drug in the formulaion; $Q\infty$: total amount of drug dissolved when the dosage form is exhausted; $K0$, $K1$, KH, Ks, Kk: release rate constants; n: release exponent

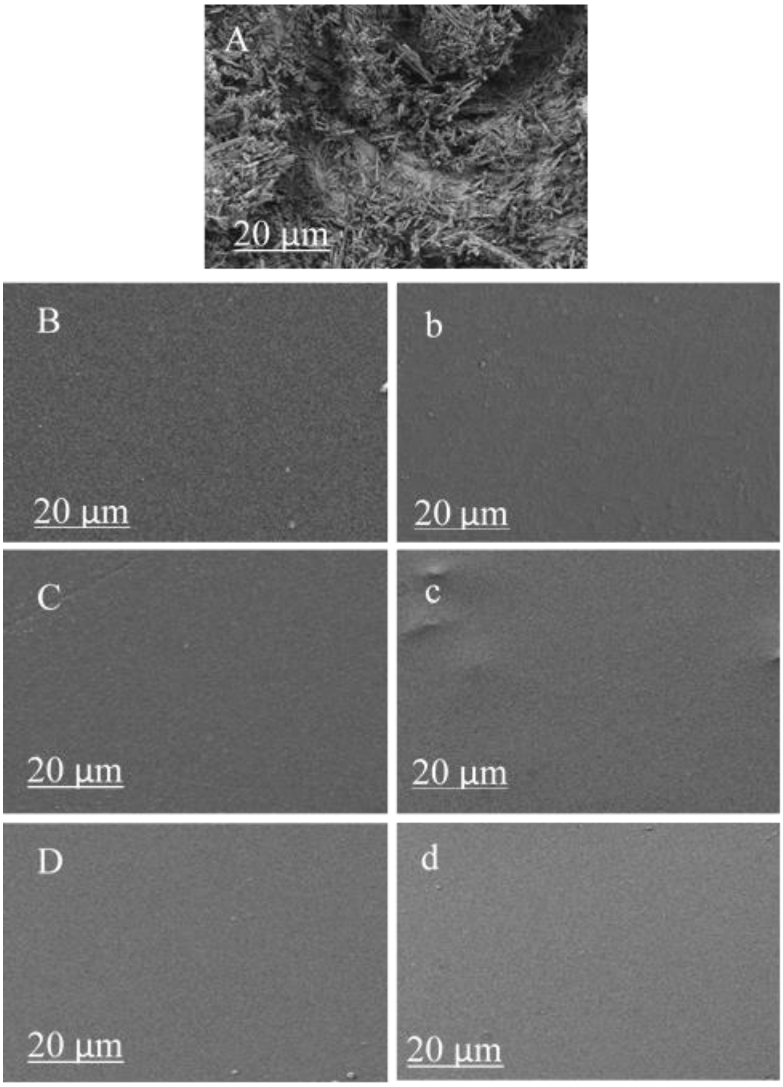

Figure S1. SEM images of (**A**) DTX, (**B**) PEVA, (**b**) PEVA $_{10}$, (**C**) PU, (**c**) PU_{10}, (**D**) Psi and (**d**) Psi_{10}.

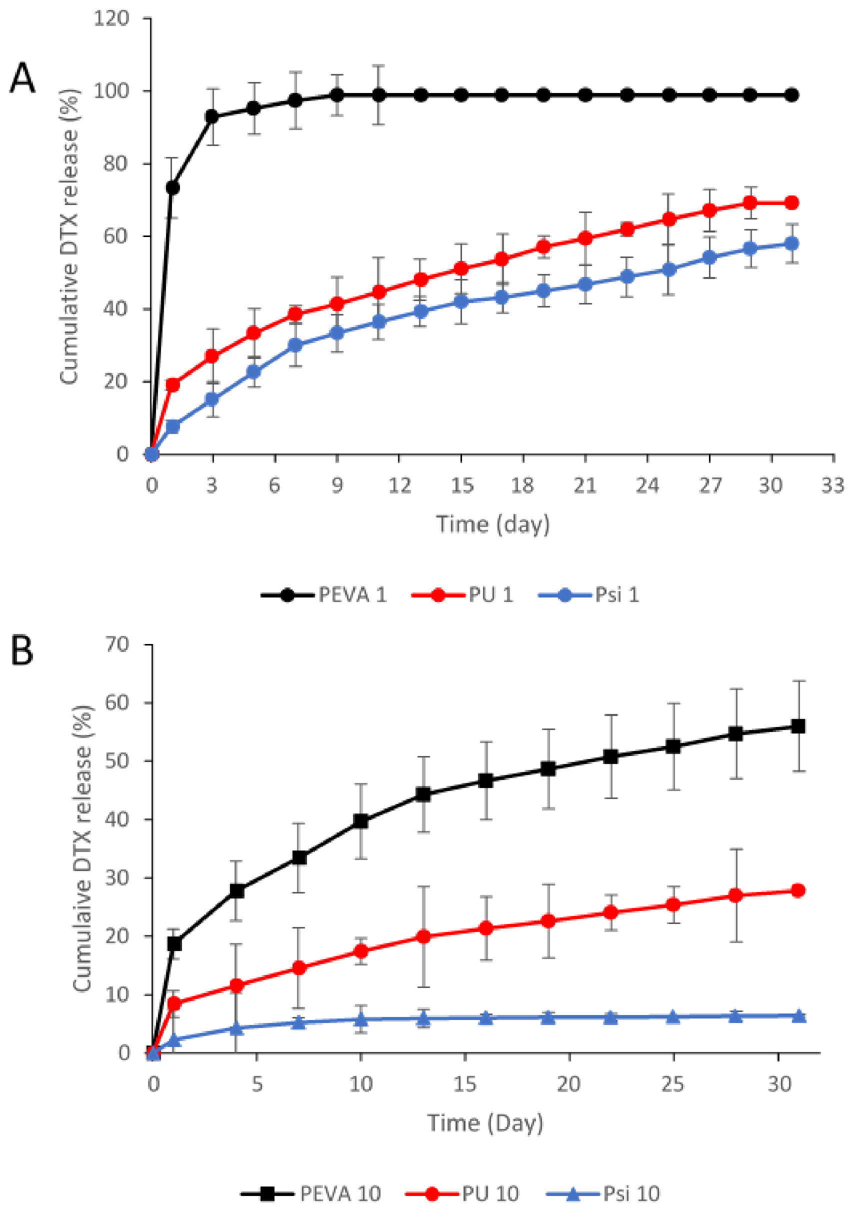

Figure S2. (**A**) In-vitro DTX release profile of $PEVA_1$, PU_1 and PS_1, (**B**) In-vitro DTX release profile of $PEVA_{10}$, PU_{10} and PS_{10}.

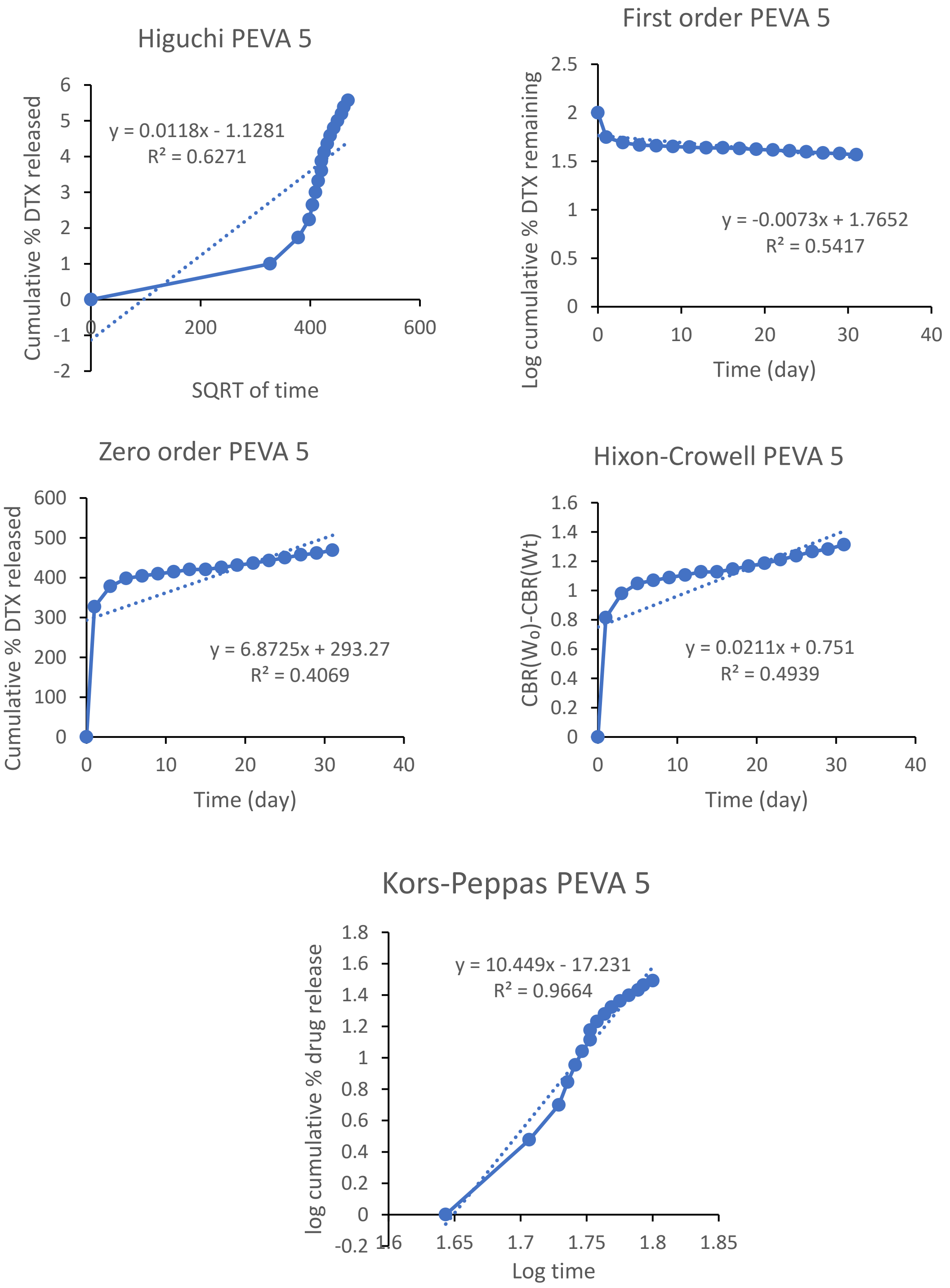

Figure S3. $PEVA_5$ in-vitro release data to Higuchi, first order, zero-order, Hixon and Kors-Peppas kinetics models.

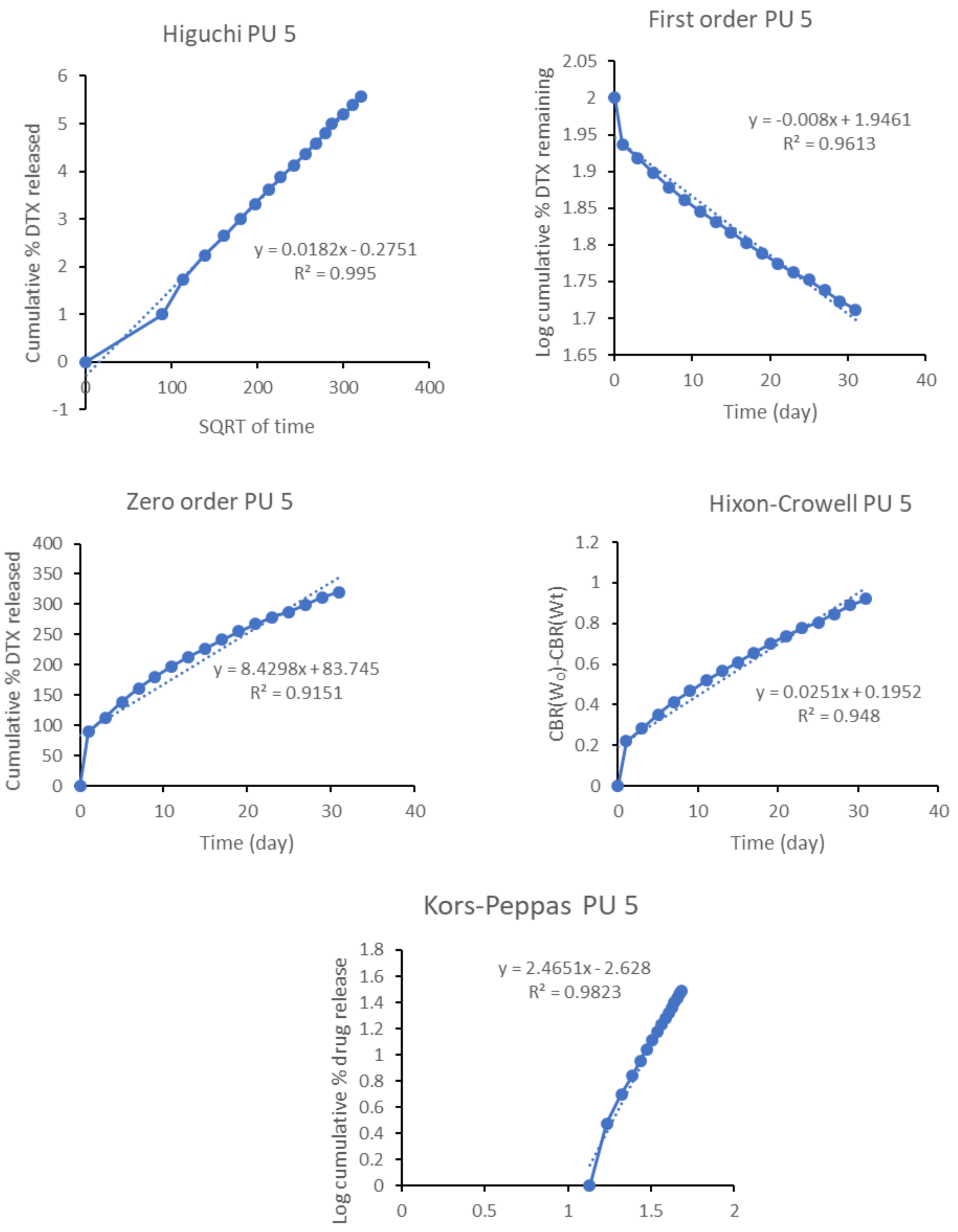

Figure S4. PU_5 in-vitro release data to Higuchi, first order, zero-order, Hixon and Kors-Peppas kinetics models.

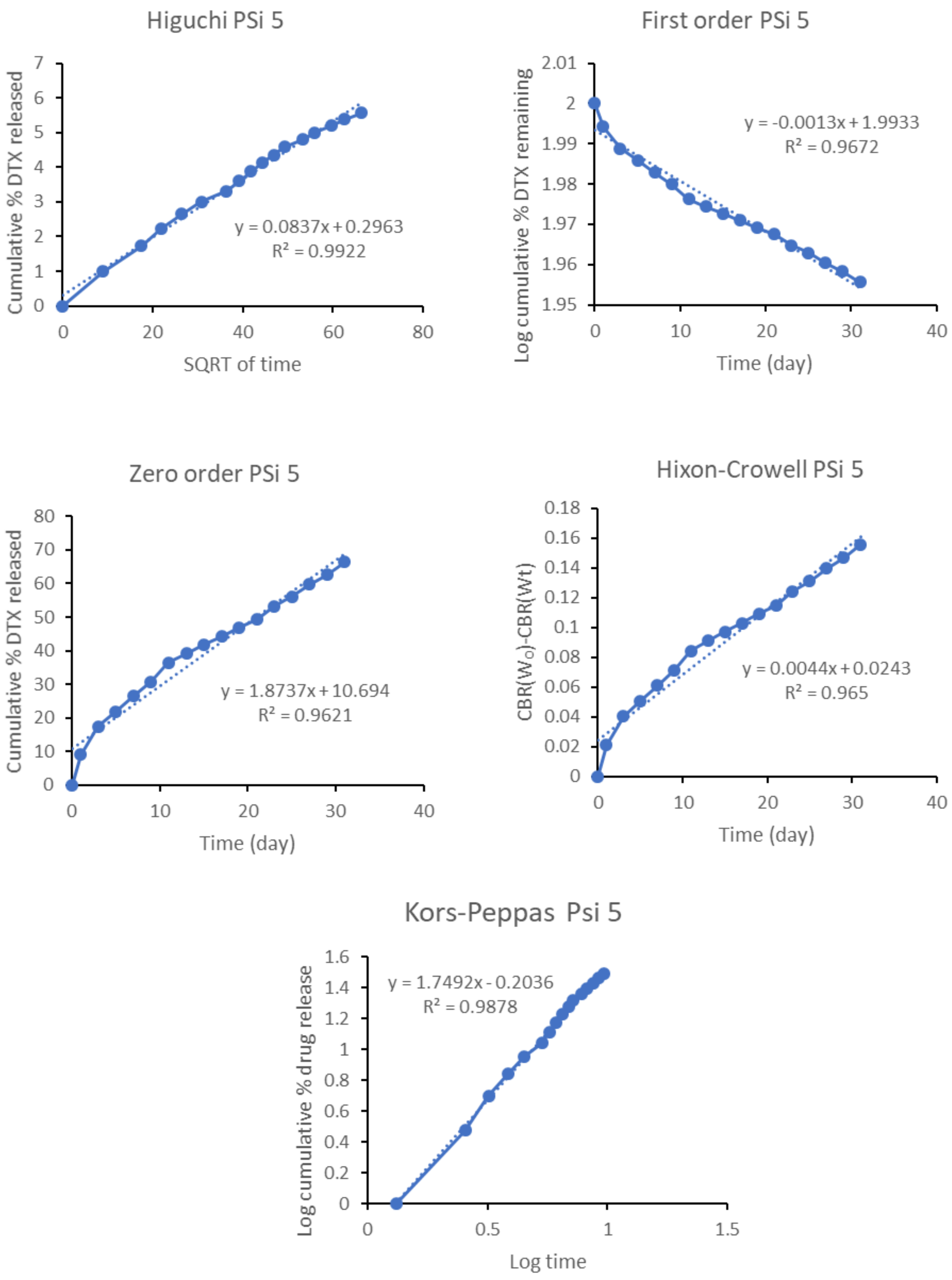

Figure S5. Psi $_5$ in-vitro release data to Higuchi, first order, zero-order, Hixon and Kors-peppas kinetics models.

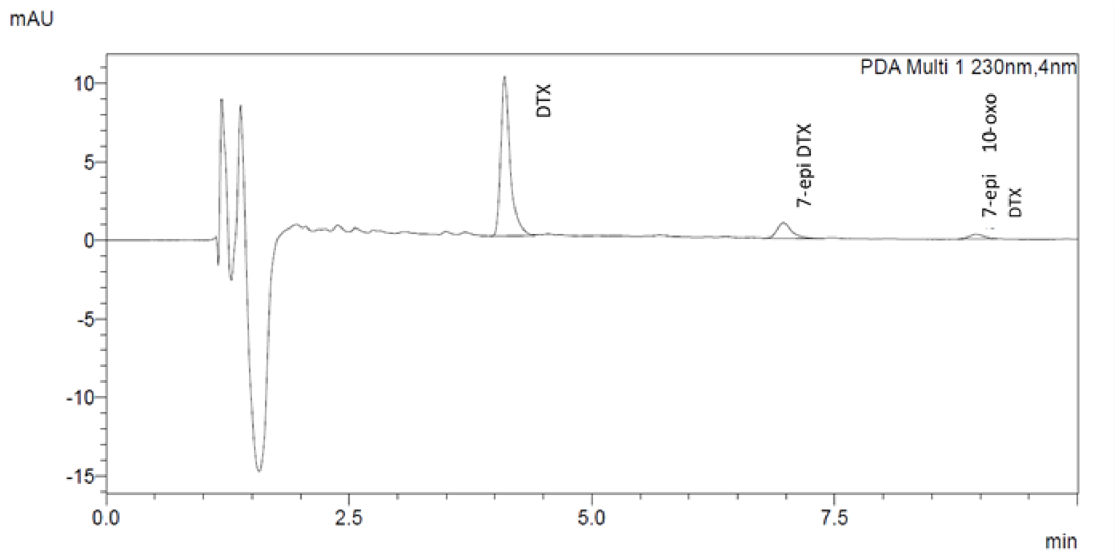

Figure S6. HPLC chromatogram of the degradation studies in presence of release media.
